# Serine/Arginine-Rich Splicing Factor 7 Knockdown Inhibits Aerobic Glycolysis and Growth in HepG2 Cells by Regulating PKM2 Expression

**DOI:** 10.3390/cimb46050301

**Published:** 2024-05-20

**Authors:** Weiye Shi, Xu Yao, Xueyu Cao, Yu Fu, Yingze Wang

**Affiliations:** College of Food Science and Biology, Hebei University of Science and Technology, Shijiazhuang 050018, China; y15203223928@126.com (X.Y.); caoxueyv1122@163.com (X.C.); fuyu@hebust.edu.cn (Y.F.)

**Keywords:** SRSF7, aerobic glycolysis, PKM2, HepG2, hepatocellular carcinoma

## Abstract

Serine/arginine-rich splicing factors (SRSFs), part of the serine/arginine-rich (SR) protein family, play a crucial role in precursor RNA splicing. Abnormal expression of SRSFs in tumors can disrupt normal RNA splicing, contributing to tumor progression. Notably, SRSF7 has been found to be upregulated in hepatocellular carcinoma (HCC), yet its specific role and molecular mechanisms in HCC pathogenesis are not fully understood. We investigated the expression and prognostic significance of SRSF7 in HCC using bioinformatics database analysis. In HepG2 cells, the expressions of SRSF7 and glycolytic enzymes were analyzed using qRT-PCR, and Western blot. Glucose uptake and lactate production were quantified using relevant reagent kits. Additionally, cell proliferation, clonogenicity, invasion, and apoptosis were evaluated using MTS assay, clonal formation assay, Transwell assay, and mitochondrial membrane potential assay, respectively. This study demonstrated significant overexpression of SRSF7 in HCC tissue, correlating with poor prognosis. Knockdown of SRSF7 in HepG2 cells resulted in inhibited proliferation, clonogenicity, and invasion, while apoptosis was enhanced. This knockdown also decreased glucose uptake and lactate production, along with a reduction in the expression of glucose transporter 1 (GLUT1) and lactate dehydrogenase A (LDHA). Furthermore, SRSF7 downregulation increased the pyruvate kinase muscle 1 (PKM1)/PKM2 ratio. The glycolytic boost due to PKM2 overexpression partially counteracted the effects of SRSF7 silencing on HepG2 cell growth. The knockdown of SRSF7 impairs aerobic glycolysis and growth in HepG2 cells by downregulating PKM2 expression.

## 1. Introduction

Hepatocellular carcinoma (HCC) represents the most prevalent primary liver cancer type, known for its high malignancy, frequent recurrence, and poor prognosis. It stands as a leading cause of cancer-related deaths globally [1]. The 2020 global cancer statistics released by the World Health Organization rank liver cancer third in global mortality and second in China [2], highlighting the critical need for new and effective therapeutic targets and strategies for HCC.

One of the hallmarks of cancer is the reprogramming of energy metabolism. Cancer cells exhibit altered metabolic pathways that support their high-energy demands and rapid growth. This metabolic reprogramming is characterized by “aerobic glycolysis”, also known as the “Warburg effect”, where cancer cells predominantly rely on glycolysis for energy production, converting most of the glucose uptake into lactate even in the presence of sufficient oxygen [3,4,5,6]. Pyruvate kinase (PK) is a key rate-limiting enzyme in glycolysis, catalyzing the transfer of a phosphate group from phosphoenolpyruvate (PEP) to ADP, resulting in the production of pyruvate and ATP [7,8]. There are four PK isoforms in mammals, including PKL (liver and kidney), PKR (red blood cells), PKM1 (muscle), and PKM2 (embryonic and cancer cells). PKL and PKR are encoded by the PKLR gene, with each isoform expressed from a tissue-specific promoter, while PKM1 and PKM2 are derived from the PKM gene through alternative splicing (AS). PKM1 lacks exon 10, while PKM2 lacks exon 9, leading to the formation of their respective mature mRNA forms. PKM2, with lower catalytic activity, facilitates the production of cellular building blocks for growth and proliferation rather than maximizing ATP production [9,10].

AS is a fundamental mechanism of post-transcriptional regulation, modulating protein function to meet the requirements of complex biological processes. However, abnormal AS can potentially contribute to carcinogenesis [11,12,13,14,15,16,17]. AS is regulated by several splicers such as SRSFs, which comprise 12 members in mammalian cells (SRSF1~12) [18,19]. Dysregulation of SRSFs can lead to abnormalities in AS, facilitating disease development, particularly cancer. Numerous studies have shown that most SRSFs exhibit abnormal expression in various tumors [20,21]. SRSF7, also known as 9G8, was identified as a member of the SR protein family in 1994 [22]. While some investigations suggest a positive role for SRSF7 in colon and lung cancer [23], its specific functions and mechanisms in HCC development remain to be elucidated.

In this study, we demonstrate for the first time that suppression of SRSF7 leads to a decrease in aerobic glycolysis and growth capacity in HepG2 cells. This reduction in aerobic glycolysis and growth is achieved by modulating the alternative splicing of PKM1 and PKM2.

## 2. Materials and Methods

### 2.1. Pancancer Analysis and Survival Analysis

RNAseq data and related clinical data for various tumor types were obtained from the Cancer Genome Atlas (TCGA) database (https://portal.gdc.com (accessed on 13 March 2023)). Statistical analysis was performed using R software v4.0.3. * *p* < 0.05, ** *p*  <  0.01, *** *p*  <  0.001.

The Kaplan–Meier Plotter online website (https://kmplot.com/analysis (accessed on 13 March 2023)) was used to analyze the relationship between gene expression levels and various clinicopathological features and the overall survival time (overall survival, OS) of patients. The following steps were followed to obtain the correlation between the expression level of the target gene and the overall survival time of the patients: “Start KM Plotter for liver cancer” was selected, the SRSF7 gene name was entered, “survival evaluation indicators” was selected, “corresponding risk factors” was selected and then “Draw Plot” was selected to generate the Kaplan–Meier curves.

Utilizing The Human Protein Atlas database (https://www.proteinatlas.org (accessed on 13 March 2023)), we analyzed the expression levels of genes in pathological and normal tissues. The steps are as follows: enter the name of the SRSF7 gene in the SEARCH column, click “Tissue”, select “Liver”, and view the immunohistochemical results of the gene in normal liver tissue; click on “Pathology” and select “Liver cancer” to view the immunohistochemical results of this gene in liver cancer tissue.

### 2.2. Culture and Cell Lines

Human HepG2 HCC cells were thawed and then cultured in Dulbecco modified Eagle′s medium (DMEM; Gibco, Thermo Fisher Scientific, Waltham, MA, USA), supplemented with 10% fetal bovine serum (FBS; Gibco, Thermo Fisher Scientific, USA) and 1% antibiotics (Solarbio, Beijing, China). The incubator conditions were controlled at 37 °C and 5% CO_2_.

### 2.3. Transfection of SiRNA and Plasmids

SiRNA targeted for reducing SRSF7 and control siRNA were obtained from Beijing Tsingke Biotech Co., Ltd. (Beijing, China). The siRNA sequences for human SRSF7 were 5′-AGGAGAGUUAGAAAGGGCUTT-3′ (SRSF7#1) and 5′-GCAUCUCCUCGACGAUCAATT-3′ (SRSF7#2). The sequence of control siRNA was 5′-UUC UCC GAA CGU GUC ACG UTT-3′. Plasmids for overexpressing PKM2 and corresponding control plasmids were sourced from MiaoLing Biology (Wuhan, China). Lipofectamine 2000 and Lipofectamine RNAiMAX transfection reagents were purchased from Invitrogen (Thermo Fisher Scientific, USA). HepG2 cells were cultured to 70% confluence on plates within 24 h. The transfection mixture, prepared by combining siRNA with either Lipofectamine RNAiMAX or Lipofectamine 2000, was incubated for 15 min at room temperature before being added to the cell cultures. After 24 h or 6 h of transfection, the initial medium was replaced with complete medium.

### 2.4. Real-Time Quantitative PCR (qRT-PCR)

RNA from HepG2 cells was extracted using TransZol up kits (TransGen Biotech, Beijing, China) and reverse-transcribed into cDNA using the TransScript One-Step gDNA Removal and cDNA Synthesis SuperMix kit (#AT-311, TransGen Biotech, Beijing, China), according to the manufacturer′s instructions. Real-time quantitative PCR was performed using the SYBR Green qPCR Master Mix (#A311, GenStar, Beijing, China). Target gene expression was normalized to β-actin and calculated using the 2^−∆∆Ct^ method. The primer sequences are listed in Table 1.

### 2.5. Western Blot Analysis

Proteins were extracted from cells using RIPA lysis buffer (Solarbio, Beijing, China) containing protease and phosphatase inhibitors. Protein concentrations were determined using a BCA protein quantification kit (Solarbio, Beijing, China). Equal amounts of protein (20 µg) were separated on 10% SDS-PAGE gels and transferred to PVDF membranes (#ISEQ00010, Millipore, Billerica, MA, USA). Membranes were then blocked in blocking buffer (TBST solution with 5% skim milk) for 1 h at room temperature and incubated with primary antibodies overnight at 4 °C. The following day, the membranes were washed. Then, the membranes were incubated with the secondary antibody (1:5000) for 1 h at room temperature. Protein bands were visualized using an ECL kit (#P0018M-2, Beyotime, Beijing, China), with β-actin serving as the internal loading control. Protein expression was analyzed using Image J software (version 2.3.0). The primary antibodies used included anti-β-actin (Proteintech, Chicago, IL, USA), anti-SRSF7 (Proteintech, Chicago, IL, USA), anti-PKM1 (Proteintech, Chicago, IL, USA), anti-PKM2 (PTM BIO, Hangzhou, China), anti-LDHA (Proteintech, Chicago, IL, USA), and anti-GLUT1 (Proteintech, Chicago, IL, USA).

### 2.6. Cell Proliferation Assay

HepG2 cell proliferation was assessed using MTS assays. Cells were seeded and transfected in 96-well plates (7.5 × 10^3^ cells/well), with 5 wells per group. At 24, 48, and 72 h post-transfection, 10 µL of MTS reagent (#G3582, Promega) was added to each well and incubated for 2 h. Absorbance (OD) at 490 nm was measured to determine cell viability.

### 2.7. Colony Formation Assay

Cells (2 × 10^5^) were seeded and transfected in 6-well plates. After 48 h of transfection, the culture medium was removed, cells were washed twice with PBS, fixed with 2% fixing solution at room temperature for 20 min, and stained with 0.1% crystal violet for 30 min. After staining, cells were washed twice with PBS, and the plates were imaged.

### 2.8. Transwell Invasion Assay

The invasion capacity of HepG2 cells was assessed using Transwell chambers (Corning, Jiangsu, China). Transfected cells (7.5 × 10^5^ cells/well) were seeded in the upper chamber and cultured in a serum-free medium, while the lower chamber contained complete medium with 10% fetal bovine serum. After 48 h of transfection, invasive cells in the lower chamber were fixed with 2% fixing solution at room temperature for 20 min and then stained with 0.1% crystal violet for 30 min. The cells were then washed with phosphate-buffered saline (PBS) (Biosharp, Beijing, China) and observed using an inverted microscope. The average number of cells in three random fields was calculated.

### 2.9. Glucose Assay

HepG2 cells were seeded in 6-well plates at 3.75 × 10^5^ cells/well. After 24 h, the cells were transfected with siRNA or plasmids for 48 h. Glucose levels were measured using a Glucose Assay Kit according to the manufacturer′s protocol (Solarbio, Beijing, China). Glucose production was normalized to the number of cells.

### 2.10. Lactate Assay

Lactate levels were measured in a similar manner, using an L-Lactate Assay Kit according to the manufacturer′s protocol (Solarbio, Beijing, China). Lactate production was normalized to the number of cells.

### 2.11. Mitochondrial Membrane Potential Assay

Mitochondrial membrane potential levels were measured using a Mitochondrial Membrane Potential Detection Kit (JC-1) according to the manufacturer′s protocol (Solarbio, Beijing, China).

### 2.12. PKM Splicing Assays

Total RNA was extracted using TRIzol and reverse transcribed using the TransScript One-Step gDNA Removal and cDNA Synthesis SuperMix (TransGen Biotech, Beijing, China). The resultant cDNA was PCR-amplified and digested using *Pst* I, followed by separation on 10% non-denaturing PAGE. Primer sequences used in the PCR reactions were as follows: human PKM exon 8 Forward: 5′-AGAAACAGCCAAAGGGGACT-3′; human PKM exon 11 Reverse: 5′-CATTCATGGCAAAGTTCACC-3′.

### 2.13. Statistical Analysis

Image J software 1.54i was used to calculate cell numbers and measure band grayscale values. All experiments were conducted in triplicate as independent experiments. Statistical analyses were performed using GraphPad Prism 8 software (GraphPad Software Inc., Boston, MA, USA). Experimental data were analyzed using *t*-tests to compare differences between two groups, and one- or two-way ANOVAs to compare multiple groups. Data are presented as the mean ± SD. A *p*-value less than 0.05 was considered statistically significant. Significance levels were denoted as * *p* < 0.05, ** *p* < 0.01, *** *p* < 0.001 or not significant (ns).

## 3. Results

### 3.1. SRSF7 Was Highly Expressed in Liver Cancer Patients and Was Associated with a Poor Prognosis

**Figure 1:**
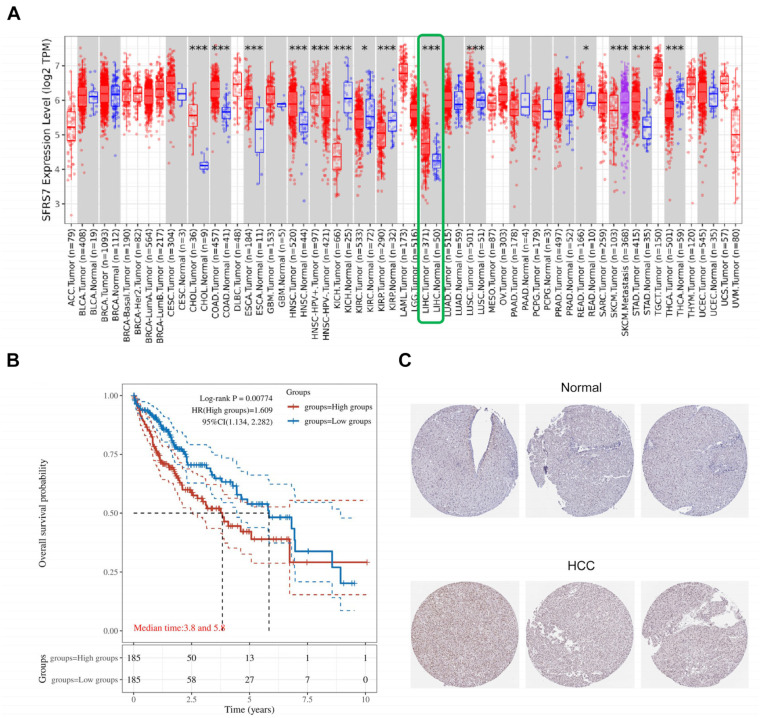
Expression and prognosis of SRSF7 in HCC. (**A**) The TIMER2.0 database was used to evaluate the differential expression of SRSF7 between various tumors and normal tissues. (**B**) Using the TCGA database, a Kaplan–Meier survival analysis was conducted. (**C**) Immunohistochemical staining of hepatocellular carcinoma tissue and normal liver tissue utilizing The Human Protein Atlas database. * *p* < 0.05; *** *p* < 0.001.

SRSF7 is abnormally expressed in various tumor tissues and plays a role in tumorigenesis. A pan-cancer analysis revealed significant differences in SRSF7 expression between hepatocellular carcinoma and normal liver tissues (Figure 1A, the red color represents tumor tissue, the blue color represents normal tissue, and the green box represents hepatocellular carcinoma), suggesting a potential role for SRSF7 in HCC development. From the TCGA database, 370 HCC patients were selected and divided into two groups based on the following SRSF7 expression levels: low SRSF7 (*n* = 185) and high SRSF7 expression (*n* = 185). Kaplan–Meier survival analysis indicated that the 10-year survival rate for patients with high SRSF7 expression was significantly lower compared to those with low SRSF7 expression. This finding implies that high SRSF7 expression might be a predictor of poor prognosis in HCC patients (Figure 1B). Immunohistochemical staining further confirmed that SRSF7 was more highly expressed in hepatocellular carcinoma tissues (Figure 1C).

### 3.2. SRSF7 Knockdown Reduces Proliferation and Invasion while Enhancing Apoptosis in HepG2 Cells In Vitro

**Figure 2:**
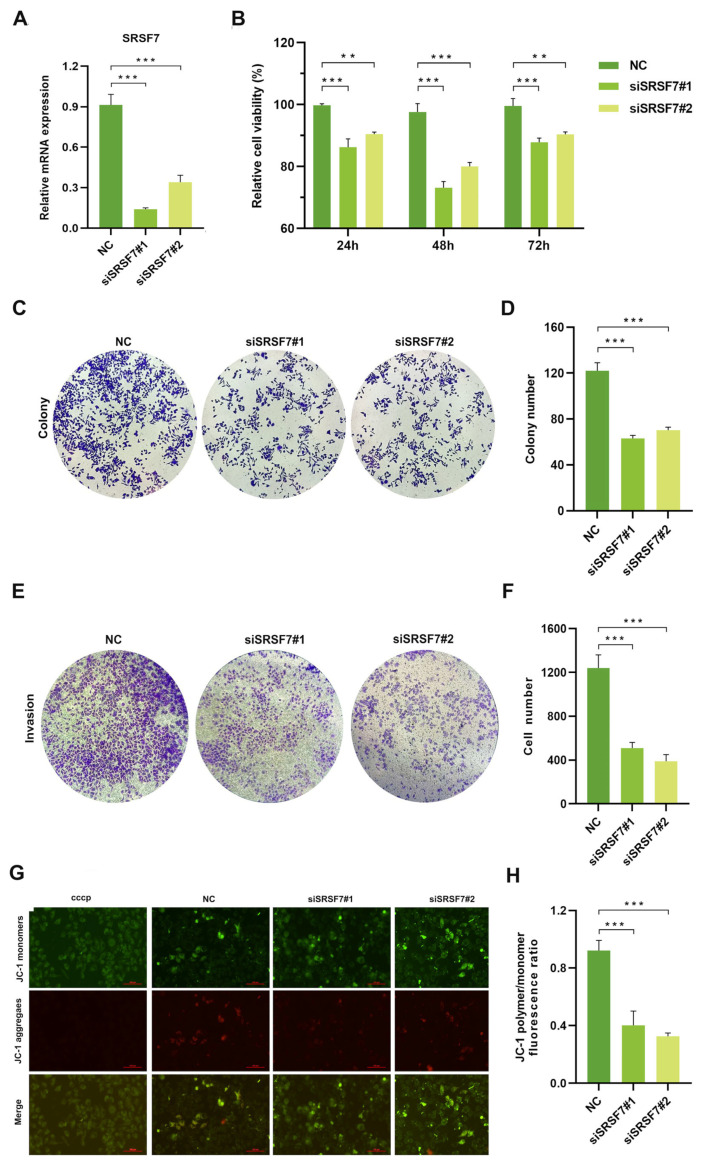
Knocking down SRSF7 alters proliferation, invasion, and apoptosis in HepG2 cells in vitro. (**A**) SiRNA-mediated inhibition of SRSF7 in HepG2 cells, with the expression levels of SRSF7 in each cell group determined by qRT-PCR. (**B**) MTS assay conducted to assess cell proliferation. The proliferation rate of HepG2 cells was significantly reduced after SRSF7 knockdown compared to the control group. (**C**) Clonal formation assay (crystal violet staining) employed to compare the clonal formation ability of cells with SRSF7 knockdown versus control cells. (**D**) Quantitative analysis of the colony counts after staining. (**E**) Transwell invasion assay (crystal violet staining) conducted 48 h after siRNA transfection to evaluate the impact of SRSF7 knockout on the invasive ability of HepG2 cells. (**F**) Quantitative analysis of colonies after staining. (**G**,**H**) Assessment of the effect of SRSF7 knockdown on cell apoptosis, as observed through inverted fluorescence microscopy, scale bar: 100 μm (**G**) and flow cytometry (**H**) in HepG2 cells. Data are presented as means ± SD (*n* = 3). ** *p* < 0.01, *** *p* < 0.001.

To evaluate the impact of SRSF7 knockdown on the proliferation, invasion, and apoptosis of HepG2 cells, we silenced SRSF7 in HepG2 cells using siRNAs. First, the efficiency of the knockdown was evaluated using qRT-PCR. The results confirmed siSRSF7 effectively silenced SRSF7 in HepG2 cells at the mRNA levels (Figure 2A). Subsequently, cell viability was assessed using the MTS assay. The findings indicated that SRSF7 knockdown significantly reduced the viability of HepG2 cells compared to the negative control group (Figure 2B). This outcome was further corroborated by a colony formation assay (Figure 2C,D). Transwell invasion assay revealed substantial inhibition in the invasive capability of HepG2 cells following SRSF7 knockdown (Figure 2E,F). A key event in early-stage apoptosis is the decline in mitochondrial membrane potential. This phenomenon was observed through the shift of JC-1 dye from red to green fluorescence, indicating a reduction in mitochondrial membrane potential. Our results showed that SRSF7 knockdown markedly promoted apoptosis in HepG2 cells, as evidenced by the change in fluorescence (Figure 2G,H). Combined, our results demonstrate that SRSF7 interference can effectively inhibit the proliferation and invasion of HepG2 cells while significantly promoting their apoptosis.

### 3.3. SRSF7 Knockdown Affects Aerobic Glycolysis in HepG2 Cells

**Figure 3:**
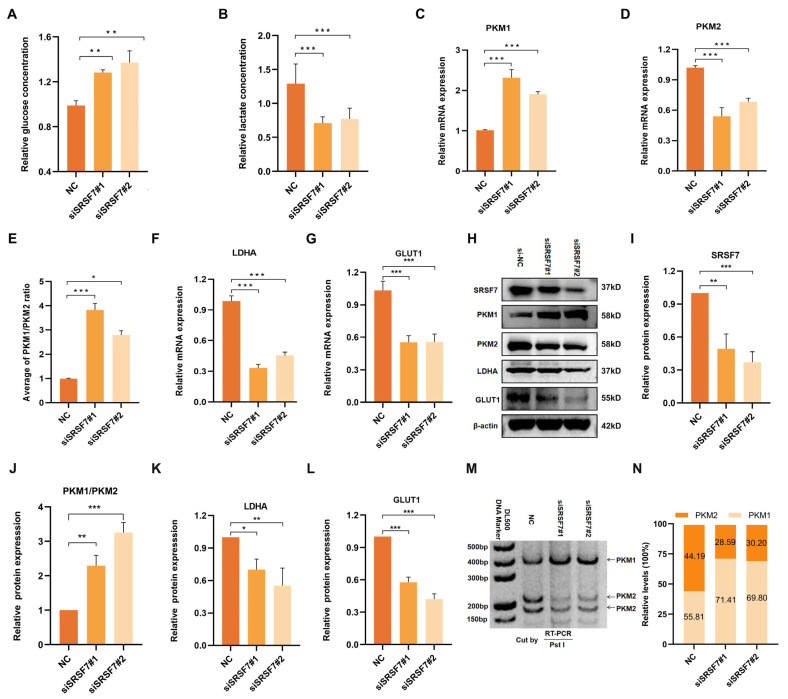
Down-regulation of SRSF7 promotes aerobic glycolysis of HepG2 cells. (A,B) Concentration of glucose in the cell culture medium (**A**) and intracellular lactate (**B**) after 48 h transfection with siSRSF7 were assessed using relevant reagent kits. (**C**–**G**) Expression levels of PKM1 (**C**), PKM2 (**D**), PKM1/PKM2 (**E**), LDHA (**F**), and GLUT1 (**G**) were determined via qRT-PCR. (**H**) Western blot analysis of SRSF7, PKM1, PKM2, LDHA, GLUT1, and β-actin in HepG2 cells after SRSF7 knockdown. (**I**–**L**) Statistical analysis of SRSF7 (**I**), PKM1/PKM2 (**J**), LDHA (**K**) and GLUT1 (**L**) protein expression levels in HepG2 cells. (**M**,**N**) PKM splicing assay after 48 h transfection with siRNA. Data are presented as means ± SD (*n* = 3). * *p* < 0.05, ** *p* < 0.01, *** *p* < 0.001.

Cancer cells often display abnormal glucose metabolism patterns, notably aerobic glycolysis, to meet their growth needs. To investigate the role of SRSF7 in the aerobic glycolysis of HepG2 cells, we measured glucose content in the cell culture supernatant and intracellular lactate content. First, we silenced SRSF7 in HepG2 cells using siRNAs. The results indicated that there was a significant increase in glucose concentration in the cell culture medium following SRSF7 knockdown (Figure 3A), suggesting a reduced glucose uptake ability in the cells. Concurrently, a decrease in intracellular lactate concentration was observed (Figure 3B), indicating a diminished capacity for lactate synthesis. These findings suggest that SRSF7 can promote aerobic glycolysis metabolism in HepG2 cells. 

Aerobic glycolysis is influenced by the abnormal expression of various transporters and key catalytic enzymes. To further explore the regulatory effects of SRSF7 on glucose metabolism in HepG2 cells, we analyzed the expression of glucose transporter 1 (GLUT1), lactate dehydrogenase A (LDHA), and two splicing isomers of pyruvate kinase (PKM1/2) following SRSF7 interference. QRT-PCR and Western blot assays were used to assess the expression of key enzymes (PKM1, PKM2, LDHA, GLUT1) in aerobic glycolysis (Figure 3C–L). Notably, PKM1 and PKM2, produced by the PKM gene via alternative splicing, play distinct roles, with PKM2 being highly expressed in most cancer cells and involved in cell proliferation and tumor formation. Our results showed that SRSF7 downregulation upregulated the expression of PKM1 (Figure 3C,H) and inhibited PKM 2 (Figure 3D,H), indicating that SRSF7 was involved in the reprogramming of glucose metabolism in HepG2 cells through the regulation of PKM alternative splicing. Additionally, the expression of LDHA (Figure 3F,K) and GLUT1 (Figure 3G,L) was downregulated following SRSF7 knockdown, aligning with the observed changes in glucose and lactate concentrations. 

We further validated the regulation of SRSF7 in the alternative splicing of PKM pre-mRNA using the splicing assay. The data showed that silencing of SRSF7 resulted in a significant increase in PKM splice-switching (Figure 3M,N). Our experimental findings demonstrate that SRSF7 influences aerobic glycolysis in HepG2 cells.

### 3.4. Verification of PKM2 Overexpression Plasmid and Assessment of Transfection Efficiency

**Figure 4:**
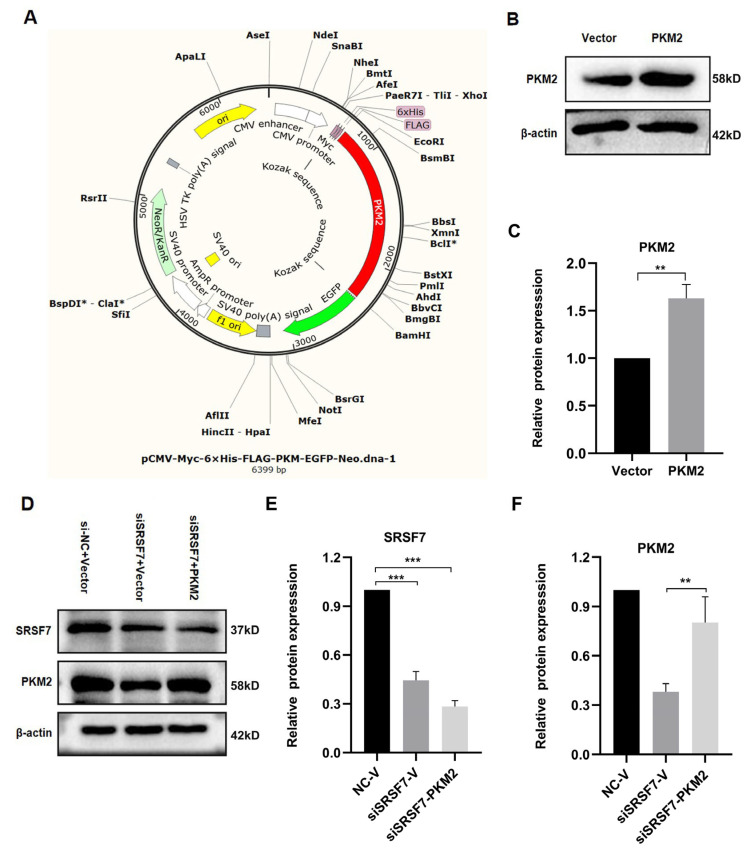
PKM2 plasmid overexpression and transfection verification. (**A**) PKM2 overexpression plasmid map. (**B**) Western blot analysis of PKM2 and β-actin in HepG2 cells after PKM2 overexpression. (**C**) Statistical analysis of PKM2 protein expression levels in HepG2 cells. (**D**) Western blot analysis of SRSF7, PKM2, and β-actin in HepG2 cells after SRSF7 knockdown and PKM2 overexpression. (**E**) Statistical analysis of SRSF7 protein expression levels in HepG2 cells. (**F**) Statistical analysis of PKM2 protein expression levels in HepG2 cells. Data are presented as means ± SD (*n* = 3). * *p* < 0.05, ** *p* < 0.01, *** *p* < 0.001.

To explore the mechanism by which SRSF7 mediates glucose metabolism reprogramming in HepG2 cells, and based on the above experimental results, we focused on the PKM2 gene for further analysis. We acquired the overexpression PKM2 plasmid pCMV-Myc-6xHis-FLAG-PKM-EGFP-Neo (Figure 4A). The PKM2 protein levels were elevated in pCMV-Myc-PKM-transfected HepG2 cells (Figure 4B,C). Co-transfection of the PKM2 plasmid with siSRSF7 for 48 h resulted in a decrease in SRSF7 protein levels, while the PKM2 protein level was increased after transfected with pCMV-Myc-PKM (Figure 4D–F).

### 3.5. Verification of PKM2 Overexpression Plasmid and Assessment of Transfection Efficiency

**Figure 5:**
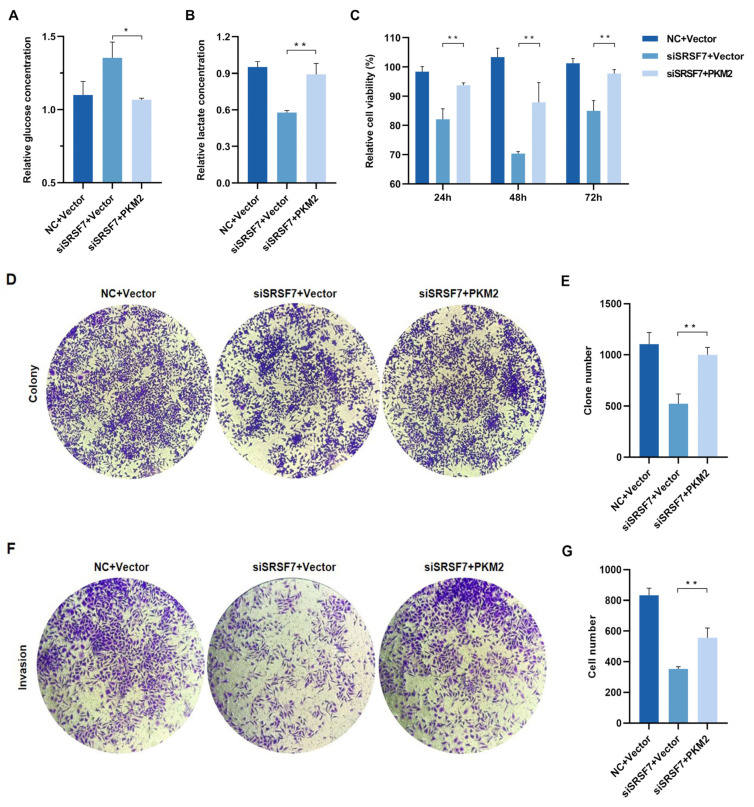
SRSF7 knockdown combined with PKM2 overexpression restore HepG2 cell aerobic glycolysis, proliferation and invasion in vitro. (**A**,**B**) After 48 h of co-transfection with SRSF7 siRNA and PKM2 overexpression plasmids, (**A**) glucose and (**B**) lactate concentrations were measured. (**C**,**D**) MTS assays (**C**) and clonal formation assays (**D**) were conducted after SRSF7 knockdown and PKM2 overexpression. (**E**) Quantitative analysis of colony counts after staining. (**F**) Transwell invasion assay was carried out after co-transfection with SRSF7 siRNA and PKM2 plasmid for 48 h. (**G**) Quantitative analysis of colony count after staining. Data are presented as means ± SD (*n* = 3). * *p* < 0.05, ** *p* < 0.01.

To reveal whether the overexpression of PKM2 reverses the effects of SRSF7 knockdown on cell aerobic glycolysis and growth, we first examined the glycolysis level, observing that both glucose consumption and lactate production in HepG2 cells increased after co-transfection with the pCMV-Myc-PKM plasmid and SRSF7 siRNA (Figure 5A,B). These results indicate that PKM2 overexpression enhances glycolysis in HepG2 cells. We further investigated if PKM2 overexpression influences the effects of SRSF7 silencing on cell proliferation and invasion. MTS and colony formation assays showed that upregulation of PKM2 expression negated the inhibitory effect of SRSF7 silencing on HepG2 cell proliferation (Figure 5C–E). Transwell invasion assays revealed that PKM2 overexpression counteracted the reduced invasion ability induced by SRSF7 knockdown (Figure 5F,G). These findings suggest that enhanced glycolysis due to PKM2 overexpression can attenuate the effects of SRSF7 silencing on HepG2 cell growth.

## 4. Discussion

**Figure 6:**
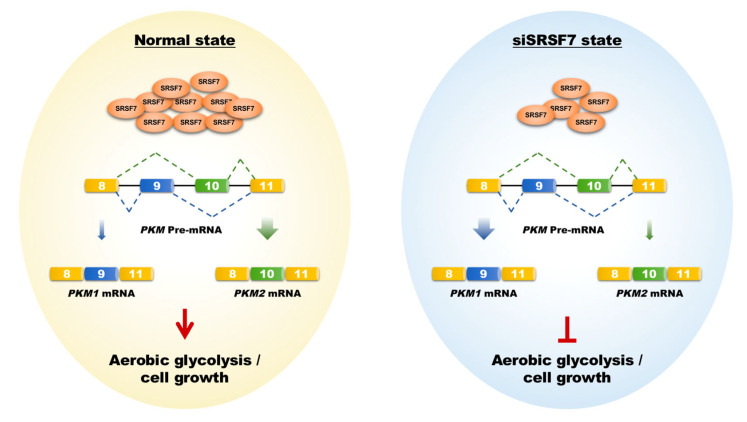
Schematic representation of how SRSF7 mediates aerobic glycolysis and cell growth in HepG2 cells via the splicing of PKM1/2. The shift from PKM2 to PKM1 splicing, induced by SRSF7 knockdown, leads to metabolic reprogramming against aerobic glycolysis, impairing cell growth and proliferation.

HCC is characterized by its insidious onset, aggressive nature, and rapid progression, making it a significant contributor to global cancer mortality [24,25]. Current molecular targeted drugs like sorafenib and lenvatinib provide some benefit in prolonging the life of advanced HCC patients. However, they are limited in preventing tumor recurrence and metastasis and are associated with notable side effects [26]. This highlights the need for novel therapeutic targets to improve treatment outcomes. In this context, our research focused on SRSF7, a splicing factor within the SR protein family. We observed a notable increase in SRSF7 expression in HCC tissues, correlating with an unfavorable prognosis. Importantly, reducing SRSF7 expression in HepG2 cells effectively suppressed aerobic glycolysis and cellular growth by downregulating the expression of PKM2 (Figure 6). 

Glucose metabolism in HCC exhibits the typical Warburg effect, supporting the rapid growth and survival of cancer cells. This metabolic shift relies on the abnormal expression of various transporters and catalytic enzymes. Initially, glucose enters cells through GLUT transporters, primarily GLUT1 and GLUT2 and is catalyzed by key enzymes like hexokinase 2 (HK2), hexokinase domain 1 (HKDC1), glyceraldehyde-3-phosphate dehydrogenase (GAPDH), and PKM2, culminating in pyruvate production via aerobic glycolysis. Pyruvate is then converted into lactate predominantly by LDHA, with monocarboxylate transporter 4 (MCT4) facilitating lactate transport to the extracellular space [27,28]. Considering this, our future research will explore whether SRSF7 also influences the splicing and biological characteristics of other catalytic enzymes. 

AS patterns in HCC include mutations in pre-mRNA splice sites, changes in regulatory elements, or alterations in splicing factors. For example, the splicing factor SRSF3 can directly bind to LNCAROD, inducing PKM isoform switching to PKM2 and enhancing aerobic glycolysis in liver cancer cells, promoting tumor malignancy and chemotherapy resistance. PTBP1, induced and recruited by RNA helicase MTR4 to pre-mRNAs of GLUT1 and PKM2, regulates target gene AS, driving metabolic changes. Under hypoxic conditions, NONO binds to mRNAs of glycolytic genes GLUT1, HK2, and LDHA, promoting their splicing maturation and increasing glycolysis levels in liver cancer cells [29,30,31]. Further investigation is necessary to determine whether SRSF7 directly acts on PKM pre-mRNA, the location of its binding sites, and the potential interactions with other splicing factors.

Based on the above research results, it is possible to explore the development of specific inhibitors targeting SRSF7 or PKM2 as novel HCC therapeutic drugs for combined use with other known anticancer drugs to improve treatment efficacy and reduce side effects. These drugs may slow down or prevent the growth and metabolism of liver cancer cells by inhibiting the function of SRSF7 or PKM2. Translating the results of this study into a clinical environment for HCC treatment has broad prospects and potential value. However, this still requires extensive research and clinical trials to validate its feasibility and effectiveness.

## 5. Conclusions

Overall, this research highlights the potential of targeting SRSF7 and PKM1/2 splice-switching as a therapeutic approach for HCC, disrupting aerobic glycolysis and inhibiting cell growth. Future in vitro and in vivo research is planned to fully elucidate the roles and mechanisms of SRSF7 in HCC.

## Figures and Tables

**Table 1:** Primers used in qRT-PCR analysis.

Gene Name	Forward Primer	Reverse Primer
β-actin	5′-GAAATCGTGCGTGACATTAA-3	5′-AAGGAAGGCTGGAAGAGTG-3
SRSF7	5′-GCGGTACGGAGGAGAAAC-3′	5′-TCGGGAGCCACAAATCAC-3′
PKM1	5′-TGAAGAACTTGTGCGAGCCT-3′	5′-GCCAGACTCCGTCAGAACTA-3′
PKM2	5′-TTACCAGCGACCCCACAGAA-3′	5′-GACGATTATGGCCCCACTGC-3′
LDHA	5′-ATGGCAACTCTAAAGGATCAGC-3′	5′-CCAACCCCAACAACTGTAATCT-3′
GLUT1	5′-TGTGCTCCTGGTTCTGTTCT-3′	5′-GCTCCTCGGGTGTCTTGT-3′

## Data Availability

Data sharing is not applicable to this article.
